# The combined role of NT-proBNP and LV-GLS in the detection of early subtle chemotherapy-induced cardiotoxicity in breast cancer female patients

**DOI:** 10.1186/s43044-021-00142-z

**Published:** 2021-03-01

**Authors:** Laila Sulaiman, Dina Hesham, Magdy Abdel Hamid, Ghada Youssef

**Affiliations:** 1 Cardiology Department, Faculty of Medicine, Cairo University, Cairo, Egypt; 2 Chemical Pathology Department, Faculty of Medicine, Cairo University, Cairo, Egypt

**Keywords:** Cardiotoxicity, Chemotherapy, Echocardiography, Brain Natriuretic peptide

## Abstract

**Background:**

Chemotherapeutic agents have many side effects; among them is cardiotoxicity. Ejection fraction fails to detect the subtle alterations of left ventricular (LV) function; that is why there is a need for a more sensitive tool. The aim is to detect subclinical LV systolic dysfunction after chemotherapeutic treatment, using NT-BNP plasma level as well as speckle tracking echo-global longitudinal strain (STE-GLS). Seventy-four asymptomatic, non-metastasizing breast cancer female patients without risk factors were included. They were assessed before and 6 weeks after taking their first chemotherapeutic session. Assessment included clinical characteristics, conventional two-dimensional (2D) and three-dimensional (3D) echocardiography, and 2D STE-GLS. Blood samples for NT-BNP plasma level were collected on both visits and were later analyzed using a Sandwich ELISA technique.

**Results:**

The median NT-proBNP almost doubled after 6 weeks of chemotherapy (73.50 vs 34.4 pg/L, p value <0.001). Only two patients showed significant reduction of LVEF >10% to less <55%. One patient died before her scheduled follow-up visit, and the cause of death is unknown. Fifty patients showed elevated follow-up levels of the NT-BNP. As compared to the baseline visit, 12 patients had a high relative reduction of the LV-GLS (>15%) and all of them had a relatively higher NT-proBNP. A 2.2 relative elevation of the NT-proBNP was able to define a relative reduction of LV-GLS >15% by a 100% sensitivity and 81.8% specificity.

**Conclusion:**

The relative reduction of LV-GLS and the relative elevation of NT-proBNP were successful in defining subclinical, subtle chemotherapy-induced cardiotoxicity after 6 weeks of the first chemotherapeutic agent administration.

## Background

Breast cancer is a major public health and economical issue; that is why research on new therapies, and monitoring their safety use, should be a priority. Globally, breast cancer is the most commonly diagnosed cancer in women, and it is the fifth leading cause of cancer death [1, 2].

In Egypt, the incidence of breast cancer among females/100,000 population of individual cancer in Lower, Middle, and Upper Egypt were 33.22%, 26.84%, and 38.72% respectively [3]. The technical advances in strategies of early detection and therapies for cancer and cancer survival have been significantly improved and cancer recurrence has been significantly reduced in the recent years. Despite this improvement in cancer therapy, however, several treatment-related adverse effects have caused serious issues for cancer survivors [4]. It is essential that specific oncologic therapies should be safe, as the life expectancy of cancer survivors is increasing.

Numerous studies had shown the cardiotoxicity of specific classes of chemotherapeutic agents. Congestive heart failure and left ventricular dysfunction are associated with the use of anthracyclines, a cumulative-dose reaction, which is more frequently seen in women with previous cardiac diseases and after mediastinal irradiation [5].

Recommendations for the diagnosis of chemotherapy-induced cardiotoxicity included functional and structural changes derived from conventional echocardiography, such as left ventricular diameters and volumes, fractional shortening (FS), and left ventricular EF (LVEF) [6, 7]. However, these conventional measurements allow only the late diagnosis of cardiac dysfunction, which by the time of diagnosis, might have been already irreversible. Hence, it is essential to find accurate and reproducible measures that could detect an early subtle LV dysfunction and, thus, be able to identify patients at risk of irreversible cardiac damage, and who may benefit from early cardioprotective measures. Global longitudinal strain (GLS) was found to be a strong predictor of cardiac dysfunction in several diseases and a reliable marker of cardiotoxicity [8].

There are several cardiac biomarkers of myocardial injury, such as troponin T (TnT), troponin I (TnI), B-type natriuretic peptide (BNP), N-terminal pro-BNP (NT-ProBNP), and myeloperoxidase (MPO). These biomarkers could detect early cardiotoxic effect of chemotherapeutic drugs after and preferably during their administration [9, 10]. Recent interest has focused on using NT proBNP plasma level to identify asymptomatic patients who are at risk of cardiovascular events. Integrated approach where GLS be combined with cardiac biomarkers could be a promising tool to define and follow up patients with chemotherapy-induced cardiotoxicity.

The aim of this study is the early detection of subtle chemotherapy-induced cardiotoxicity, using echocardiography LV-GLS and NT-proBNP plasma level.

## Methods

### Study population

This is a non-randomized, observational cohort study that included 74 asymptomatic female patients who were planned to receive chemotherapy for breast cancer in the period between November 2017 and July 2018. Exclusion criteria included a history of coronary artery disease, a hemodynamically significant valvular heart disease, and a previous chemotherapy or chest radiotherapy. Patients with known cardiovascular risk factors (old age >65 years, hypertension, diabetes, dyslipidemia, smoking, and obesity (BMI more than 30 kg/m^2^)) as well as patients with abnormal baseline LVEF (less than 50%) were also excluded.

This study complies with the Declaration of Helsinki (2013), and the Faculty of Medicine, Cairo University ethics committee has approved the research protocol. A written informed consent has been obtained from the patients.

### Methods

#### *The baseline visits*

All patients were assessed clinically and this included oncological history, type and site of cancer, a family history of breast cancer, body weight and height measurements, body mass index (BMI) {BMI was calculated according to the following formula; BMI = body weight (kg)/height (m^2^)}, and heart rate and blood pressure (BP) measurements (BP was measured using the mercury sphygmomanometer).

Some data were derived from the patients’ files and the pathology report like the TNM staging, the identified receptors (progesterone, estrogen or HER receptors), the chemotherapeutic agents used (cyclophosphamide, doxorubicin, epirubicin, and docetaxel), and the doses to be given. The cardiotoxic effects have been reported with doxorubicin doses >400 mg/m^2^, epirubicin doses >900 mg/m^2^, and cyclophosphamide doses >140 mg/kg [11].

A twelve-lead ECG was done via the department machine SCHILLER, AT-101, looking out for changes in voltage, PR interval, QRS duration, QTc interval using Bazett’s formula [12] (normal corrected QT for females is 350–440 ms) [13], any arrhythmias, or any other pathological abnormalities.

Laboratory workup included complete blood count (CBC) and kidney function tests (serum creatinine and blood urea nitrogen).

#### * NT-pro BNP level assessment

It was measured using the enzyme-linked immunosorbent assay (ELISA) technique (the kit catalogue number is E-EL-H0902) [14]. Three milliliters of blood was withdrawn from an arm vein of the patient; samples were left to clot for 2 h at room temperature or overnight at 4°C before centrifugation for 15 min at 1000×*g* at 2~8 °C. The supernatant was collected for the assay and stored at – 20 °C or – 80 °C till analysis [14].

#### * Two-dimensional (2D) transthoracic echocardiography (TTE)

Resting conventional 2D-TTE was done by a single experienced operator using a commercially available machine (Philips IE-33), equipped with a 2.5 MHz transducer, where a 2D, M-mode and Doppler images were digitally recorded for subsequent analysis. Measurements included LV dimensions and volumes, LVEF (apical biplane modified Simpson’s method), left atrial volume index, mitral inflow Doppler parameters, and mitral lateral annulus tissue Doppler (TDI) values. The Tei index was calculated by the following formula: (IVCT+IVRT/ET), where IVCT is the isovolumetric contraction time (the time from closure of the mitral valve till the opening of the aortic valve), IVRT is the isovolumetric relaxation time (the time from closure of the aortic valve till the opening of the mitral valve), and the ET is the ejection time (the time from opening till closure of the aortic valve).

Loops of the different apical views (two (A2), four (A4), and three (A3) chamber views) were stored for offline analysis of STE and GLS using a (Q-LAB 10.0) program. A good quality ECG was ensured during recording as a prerequisite for a proper speckle tracking analysis. LV longitudinal strain parameters were measured from the apical views, and the myocardium was divided into 6 segments per view (basal, mid and apical segments for the 2 walls in each view). A2-GLS, A3-GLS, and A4-GLS were reported. The overall GLS was automatically calculated as the average of the 18 segments measured in the 3 apical views.

#### * Three-dimensional (3D) TTE

All patients underwent 3D echocardiography by an experienced operator using Philips IE-33 machine equipped with an xMATRIX transducer. Within a single breath-hold, 4 wedge-shaped sub-volumes were acquired from an apical view to create full-volume data sets. Care was taken to include the entire LV within the 3D scan volume by decreasing the depth and sector width as much as possible to improve the temporal and spatial resolution of the images.

Quantitative analysis was done by using (Q-LAB 10.0) software. A semi-automated border detection biplane LV analysis was performed. For the quantification of LV volumes and function, the longitudinal axes were aligned in the first frame of the loop which corresponds to LV end-diastole in both the apical four-chamber and two-chamber views. Care was taken for the proper definition of both apical views and orthogonal views to avoid foreshortening. Then, tracing was performed by marking five points: septal, lateral, anterior, and inferior points of the mitral annulus and a fifth point on the LV apex. A semi-automated blood–endocardial interface detection algorithm then automatically identified the endocardial border and calculated the LV end-diastolic volume (LVEDV). Unsatisfactory delineation of the endocardial border was manually adjusted. The end-systole was selected by visually identifying the frame with the smallest LV cavity size just before mitral valve opening where tracing was repeated in the same manner as for the end-diastole to obtain the LV end-systolic volume (LVESV). The software automatically detects the endocardial borders in the three dimensions, throughout the cardiac cycle and calculates LV volumes and EF [15–17].

##### The follow-up visits

Patients were asked to follow up after 6 weeks of the 1st chemotherapy session. Patients went through routine clinical assessment, searching for symptoms and signs of heart failure.

Electrocardiography was done and analyzed for any changes (compared to the baseline ECG). Echocardiographic assessment (conventional 2D, 2D-STE, and 3D echocardiography) was repeated, and the same study parameters were reported). Blood samples were collected for NT-pro BNP plasma level using the same ELISA technique.

### End points

The primary end point for this study was the early detection of left ventricular systolic dysfunction, based on elevated NT-pro BNP plasma levels as well as reduction of the LV-GLS in breast cancer patients receiving chemotherapy.

Cardiotoxicity is defined as a decrease of LVEF from baseline values by >5% to an EF <55% (in the presence of heart failure (HF) symptoms and signs) or an asymptomatic decrease in LVEF by > 10% to an EF < 55% [18, 19]. Abnormal LV-GLS is defined by an absolute value less than 20 (less negative values) and/or a relative reduction of GLS > 15% of the baseline values [19].

### Statistics

Data was analyzed using Statistical Package of Social Science (SPSS) version 20. Categorical data are described as numbers and percentages and continuous data are described as means and SD or median and range. A paired sample Student’s *t* test (for data that was normally distributed) or a Mann–Whitney test (for data that was not normally distributed) was used to compare data at the baselines and follow-up visits. Spearman correlation test was used to define the degree and direction of the relationship between the delta change of LV GLS and that of NT-proBNP plasma levels. The delta values of the NT-proBNP, 2D-LVEF, 3D LVEF, and 2D LV-GLS were calculated as the follow-up value minus the baseline value. The relative change of the GLS and the NT-proBNP were calculated as follows ({follow-up value − baseline value}/baseline value). A multivariate linear regression analysis was used and age, laboratory data, and echocardiographic data were introduced into the regression model to identify the independent predictors of the delta change of NT-proBNP level as well as the delta change of the GLS. A receiver operator characteristic (ROC) analysis was used to detect the relative change of NT-proBNP that was capable of defining a 15% relative change of the GLS with a good sensitivity and specificity. Two-tailed *p* value <0.05 was considered significant.

## Results

### The baseline clinical characteristics (Table 1)

Seventy-four female patients were included in our sample, the minimum age of whom was 24 and the maximum was 65 years, and 9 patients (12%) were older than 60 years.

**Table 1:** Baseline (first visit) clinical characteristics of the patients

Variables	No. (%)
Age in years, mean ± SD	47.6 ± 10.5
FH of cancer	3 (4.1)
**Type of receptors**	
Progesterone receptor (PR) positive	48 (64.9)
Estrogen receptors (ER) positive	22 (29.8)
HER receptor positive	61 (82.5)
**TNM staging**^a^	
**T (tumor size)**	
T1	7 (9.5)
T2	46 (62.2)
T3	21 (28.4)
**N (lymph node spread)**	
N0	18 (24.3)
N1	56 (75.7)
**M (blood spread)**	
M0	74 (100)
**Grade of breast cancer**	
IDC-II^b^	71 (95.9)
IDC-III^b^	3 (4.1)
**Site of breast cancer**	
Right	32 (43.2)
Left	41 (55.4)
Bilateral	1 (1.35)
**Surgery**	
Pre chemotherapy	54 (73.0)
Post chemotherapy	20(27.1)
**Planned chemotherapeutic agents**	
A (doxorubicin)	43 (58.1)
E (epirubicine)	27 (36.5)
T (docetaxel)	4 (5.4)
C (cyclophosphamide)	73 (98.6)

*DM* diabetes mellitus, *HTN* hypertension, *IDC* invasive ductal carcinoma, *FH* family history, *T* tumor, *N* lymph node, *M* metastasis

^a^TNM staging where T refers to the tumor size (T1; size <2 cm, T2; size 2–5 cm, T3; size >5 cm), N refers to lymph node affection (N0; no cancer cells in the nearby lymph nodes, N1; some cancer cells are found in the axillary lymph nodes, but the nodes are not stuck to the surrounding), and M refers to blood metastases (M0; no metastases)

^b^IDC, invasive ductal carcinoma (grade II is a moderately differentiated while grade III is a poorly differentiated IDC)

Twenty patients (27.1%) were planned to receive chemotherapy before surgery (radical mastectomy or tumor resection), and thus, their follow-up was done before surgery.

Almost all patients had received anthracycline-type chemotherapy, with the mean cumulative dose of doxorubicin, at the FU visit = 312 mg/m^2^ (3 cycles) while the mean cumulative dose of epirubicin was 405 mg/m^2^ (3 cycles), none of the mean cumulative doses exceeded the cardiotoxic range for doxorubicin or epirubicin.

### Comparison between baseline and follow-up visits

In the follow-up (FU) visit, all patients, but one, had completed three sessions of their chemotherapy. None of the patients had heart failure symptoms or signs. Eleven patients (14.9%) developed marked hemoglobin drop but only 2 of them needed blood transfusion. Two other patients (2.7%) suffered from low leukocytic count that necessitated delaying of the next chemotherapeutic session. One patient died, before her follow-up visit. She was 50-year-old with IDC II in the left breast T3N1M0 and positive HER2 and PR receptors. She received two cycles of epirubicin (110 mg/cycle) and cyclophosphamide (1100 mg/cycle) with surgery being planned post chemotherapy. Her baseline NT-pro BNP was 48 pg/ml. She had a normal clinical, ECG and echocardiographic parameters before chemotherapy. The cause of her death is unknown.

Table 2 shows that the follow-up systolic blood pressure (SBP), hemoglobin (Hb), and platelets were significantly lower, while the follow-up heart rate was significantly higher when compared to the baseline values, yet all remained within the normal range.

**Table 2:** Clinical parameters and laboratory results in the baseline and follow-up visits

Variables	Baseline (mean± SD)	FU (mean± SD)	***P*** value
SBP (mmHg)	118.9 ± 8.5	115.8 ± 7.5	**0.001**
DBP (mmHg)	71 ± 7.8	69.1 ± 8.1	0.084
HR (bpm)	81.4 ± 12.3	85.2 ± 13.7	**0.012**
Weight (kg)	72.4 ± 9.7	71.4 ± 9.4	0.117
BMI (kg/m^2^)	26.4 ± 2.7	26.2 ± 2.61	0.256
Hb (g/dl)	12.8 ±0.8	12.1 ± 1.1	**<0.001**
Platelets (× 10^9^/L)	348.5 ± 98.9	333.3 ± 100.6	**0.007**
Creatinine (mg/dl)	0.8 ± 0.1	0.8 ± 0.1	0.779
NT-pro BNP, median (range), pg/ml	34.4 (446.2)	73.5 (956.4)	**<0.001**
Delta NT-pro BNP, median (range), pg/ml	16.2 (535.6)		

*SBP* systolic blood pressure, *DBP* diastolic blood pressure, *BMI* body mass index, *HR* heart rate, *Hb* hemoglobin

The median NT-proBNP remained within the normal range, yet almost doubled in the FU visit, as demonstrated in Table 2. Fifty patients (67.6%) had higher follow-up NT-proBNP levels (compared to the baseline values) with a minimum difference of 3.1 pg/ml and a maximum of 518 pg/ml. The remaining 23 patients (one sample was missing as one patient died before her FU visit) had a reduction in their follow-up NT-proBNP levels with a minimum difference of − 1.5 pg/ml and a maximum reduction of − 17 pg/ml.

### Echocardiographic results (Table 3)

In the follow-up visit, the 3D-LVEF was significantly lower (with a mean difference of − 2.0%) while the LVESV (by 2D and 3D) as well as the Tei index were significantly higher as compared to the baseline levels, yet all remained within the normal range. None of the patients had left ventricular systolic dysfunction (by absolute 2D-EF values). The mean follow-up TAPSE and S-wave TDI TV velocity were high yet all were within the normal ranges (Table 3).

**Table 3:** Conventional 2D and 3D echocardiographic data of the study patients in the baseline and follow-up visits

Parameters	Baseline (mean± SD)	FU (mean± SD)	***P*** value
M-Mode LVEF (%)	64.6 ± 5.60	62.5 ± 6.98	**0.028**
LVFS (%)	35.3 ± 4.83	34.12 ± 5.80	0.174
LVDD (cm)	4.5 ± 0.53	4.61 ± 0.60	0.103
LVSD (cm)	2.91 ± 0.40	3.54 ± 4.12	0.189
SWT (cm)	0.84 ± 0.11	0.83 ± 0.11	0.584
PWT (cm)	0.9 ± 0.10	0.9 ± 0.11	0.887
**2D volumes**			
LVEDV (ml)	62.51±17.10	64.7±17.81	0.373
LVESV (ml)	23.8±6.93	26.2±8.14	**0.029**
LVEF (%)	61.7±5.10	60.32±5.60	0.115
Mean delta 2D LVEF (%)	− 1.4±7.3		
LAVI (mL/m^2^)	20.24±5.44	19.21±4.50	0.052
**Diastolic function**			
E (cm/s)	0.8±0.17	1.71±7.9	0.323
A (cm/s)	0.65±0.21	0.7±0.17	0.365
E/A ratio	1.32±0.39	1.24±0.39	**0.049**
EDT (ms)	182.14±33.99	183.1±23.20	0.839
S TDI MV (cm/s)	11.7 ±9.15	10.9±1.90	0.435
E’ (cm/s)	11.65 ±3.13	11.7±3.05	0.924
A’ (cm/s)	9.31 ±3.10	10.12±2.62	**0.028**
E/E’ ratio	6.96±2.40	6.9±2.40	0.823
Tei index	0.31±0.10	0.34±0.10	**0.014**
**RV function**			
TAPSE (cm)	2.5±0.49	2.8±0.47	**<0.001**
FAC (%)	51.51±7.73	51.3±7.90	0.808
PASP mmHg	3.44±8.10	3.5±8.04	0.873
S-TDI TV (cm/s)	13.23 ±2.03	13.75±2.02	**0.027**
RVOT (cm)	1.7±0.40	1.7±0.32	0.570
CS (cm)	0.72±0.15	0.73±0.12	0.572
IVC (cm)	1.4 ±0.33	1.41 ±0.29	0.959
**3D Echocardiography**			
LVEDV (mL)	74.34±15.14	75.94 ±18.99	0.523
LVESV (mL)	28.13±6.32	30.38±9.05	**0.037**
EF (%)	61.84 ±5.79	59.88±5.42	**0.031**
Mean delta 3D LVEF (%)	− 2.0 ± 7.4		

*LVEF* left ventricular ejection fraction, *LVFS* left ventricular fractional shortening, *LVDD* left ventricular diastolic dimension, *LVSD* left ventricular systolic dimension, *SWT* septal wall thickness, *PWT* posterior wall thickness, *2D* 2 dimensional, *LVEDV* left ventricular end diastolic volumes, *LVESV* left ventricular end systolic volumes, *LAVI* left atrium volume indexed, *EDT* E deceleration time, *TDI* tissue Doppler imaging, *MV* mitral valve, *RV* right ventricle, *TAPSE* tricuspid annular plane systolic excursion, *FAC* fractional area change, *PASP* pulmonary artery systolic pressure, *TV* tricuspid valve, *RVOT* right ventricular outflow tract, *CS* coronary sinus, *IVC* inferior vena cava

Only two patients showed relative reduction of the EF >10% to less than 55% (patients’ numbers 52 and 57) using a 2D biplane Simpson’s method and both of them had elevated follow-up NT-proBNP (with relative values are 1.9 times and 1.7 times the baseline values respectively). Using 3D images, only one patient fulfilled the criteria of reduction of LVEF (patient 57 had a follow-up 3D LVEF that was 15% lower than the baseline with an absolute value of 54.4%).

In concordance with the 2D echocardiography results, 3D LVESV was significantly higher and the EF was significantly lower in the follow-up visit compared to baseline visit, yet remained within the normal range.

### Speckle tracking data (Table 4)

Only 61 patients (82.4%) had eligible echocardiographic images for STE analysis (Table 4). There were 12 patients (19.7%) who showed relative reduction of GLS >15%, of whom, 8 patients (66.7%) had an abnormally low GLS (less negative than − 20). Overall, 26 patients (42.6%) showed an abnormal GLS in the follow-up visit.

**Table 4:** Comparison of the baseline and FU levels of GLS

Variable	Baseline (mean SD)	FU (mean SD)	***P*** value
A4GLS (%)	− 22.0 ± 2.6	− 20.1 ± 2.6	<0.001
Mean delta A4GLS	− 1.9 ± 2.1		
A3GLS (%)	− 21.0 ± 2.6	− 19.2 ± 2.7	<0.001
Mean delta A3GLS	− 1.8 ± 2.9		
A2GLS (%)	− 21.6 ± 3.2	− 19.6 ± 2.8	<0.001
Mean delta A2GLS	− 2.0 ± 2.9		
Average GLS (%)	− 21.5 ± 2.3	− 19.7 ± 2.1	<0.001
Mean delta average GLS	− 1.8 ± 1.8		

*A4GLS* apical 4-chamber view global longitudinal strain, *A3GLS* apical 3-chamber view global longitudinal strain, *A2GLS* apical 2-chamber view global longitudinal strain

**Fig. 1:**
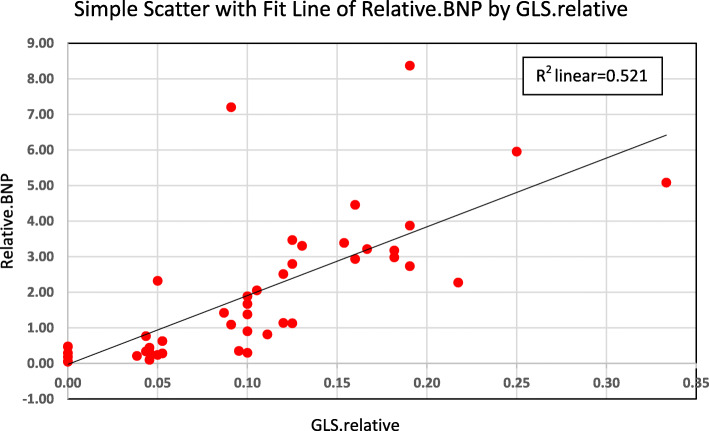
The correlation between the relative change of NT-proBNP and the relative change of LV-GLS (*r* = 0.833 and *p* < 0.001)

The median NT-proBNP was higher in patients with abnormally low (less negative) GLS (*n*=26) in the FU visit compared to patients with normal GLS (92.75 vs 73.5 pg/ml, *p*=0.375). On the other hand, the median NT-proBNP levels were significantly higher in patients with bigger (>15%) relative reduction of GLS compared to patients with less relative reduction of GLS (137.2 vs 66.0, *p* < 0.001). Figure 1 shows the strong positive correlation between the relative change of the 2D-GLS and the relative change of the NT-proBNP plasma level (*r* = 0.833, *p* < 0.001).

A comparison was made between patients who had elevated follow-up NT-proBNP (*n* = 50) and those who did not (*n* = 23) (Table 5). The former patients showed a significant reduction in their 2D and 3D LVEF and as well as their 2D-GLS.

**Table 5:** Comparing patients who had lower versus those who had higher NT-proBNP at the follow-up visit

Variables	Patients with lower FU NT-proBNP (*n* = 23)			Patients with higher FU NT-proBNP (*n* = 50)		
	Baseline	FU	*p* value	Baseline	FU	*p* value
2D-LVEF	61.3 ± 5.8	62.5 ± 7.1	0.47	61.8 ± 4.7	59.3 ± 4.4	0.012
Mean delta 2D-LVEF	1.2 ± 7.7			− 2.5 ± 6.9		
3D-LVEF	60.6 ± 5.6	60.3 ± 6.7	0.87	62.4 ± 5.8	59.0 ± 6.4	0.01
Mean delta 3D-LVEF	− 0.3 ± 8.3			− 2.7 ± 6.9		
2D LV-GLS	− 21.0 ± 1.9	− 20.1 ± 1.7	0.055	− 21.7 ± 2.4	− 19.4 ± 2.2	<0.001
Mean delta 2D LV-GLS	− 0.3 ± 0.6			− 2.3 ± 1.8		
Median NT-proBNP, (range), pg/ml	37.5 (66.0)	32.1 (62.9)	<0.001	23.7 (446.2)	91.5 (949.5)	<0.001
Median NT-proBNP change (range), pg/ml	− 5.5 (15.6)			46.1 (515.4)		

*FU* follow-up, *LVEF* left ventricular ejection fraction, *GLS* global longitudinal strain

Out of the 50 patients who had elevated NT-proBNP levels in the FU visit, only 5 patients had markedly elevated follow-up NT-proBNP levels (>200 pg/ml). Those 5 patients had IDC II, pre-chemotherapy surgery, and all were positive in HER 2 receptors (Table 6). There were no significant ECG or lab abnormalities in those patients. Patient number 19 was the only patient with a high baseline NT-proBNP. There was no clinical or echocardiographic explanation for her elevated NT-pro BNP level at baseline, except for the abnormally low baseline LV-GLS (− 16).

**Table 6:** Demographical, oncological, and echocardiographic data of patients who had an abnormally elevated level of plasma NT-pro BNP in the follow-up visit

Patients no.	Pt.7	Pt.12	Pt.13	Pt.19	Pt.38
Age (years)	53	65	47	60	58
Site of cancer	R	L	L	R	R
FH of cancer	YES	NO	NO	NO	NO
Type of cancer	IDC II	IDC II	IDC II	IDC II	IDC II
TNM staging	2,1,0	2,1,0	2,0,0	2,1,0	2,1,0
Progesterone receptors	Positive	Positive	Positive	Negative	Negative
Estrogen receptors	Negative	Negative	Positive	Negative	Negative
HER2	Positive	Positive	Positive	Positive	Positive
Chemotherapy type 1	A	E	E	A	E
Dose in mg/cycle	110	100	160	90	150
Cumulative dose in mg (after 3 cycles)	330	300	480	270	450
Chemotherapy type 2	C	C	C	C	C
Dose in mg per cycle	1100	1000	1200	900	1000
Cumulative dose in mg (after 3 cycles)	3300	3000	3600	2700	3000
Timing of surgery	Before chemotherapy	Before chemotherapy	Before chemotherapy	Before chemotherapy	Before chemotherapy
2D LVEF (baseline)	57	64	60	60	59
2D LVEF (FU)	64	54	59	58	55
**Relative change of 2D LVEF**^a^	**+ 0.12**	**− 0.16**	**− 0.02**	**− 0.03**	**− 0.07**
2D-GLS (Baseline)	**−** 21	**−** 20	**−** 22	**−** 16	**−** 27
2D-GLS (FU)	**−** 17	**−** 15	**−** 18	**−** 14	-18
**Relative change of 2D-GLS**^a^	**− 0.19**	**− 0.25**	**− 0.18**	**− 0.13**	**− 0.33**
3D LVEF (Baseline)	55.8	57.7	60.2	60.7	69.0
3D LVEF (FU)	56.0	57.0	60.0	62.0	59.0
**Relative change of 3D-LVEF**^a^	**+0.004**	**− 0.012**	**− 0.003**	**+0.021**	**− 0.145**
NT-pro BNP, pg/ml (Baseline)	36.5	66	81.5	459	34.3
NT-pro BNP, pg/ml (FU)	342	459	324.6	977.5	208
**Relative change of NT-proBNP**	**+8.4**	**+6.0**	**+3.0**	**+1.1**	**+5.1**

*FH* family history, *TNM* tumor lymph node metastasis, *HER2* human epidermal growth factor receptor 2, *CH* chemotherapy, *R* right, *L* left, *IDC* invasive ductal carcinoma, *A* doxorubicin, *E* epirubicin, *C* cyclophosphamide, *PRE* surgery before chemotherapy

^a^Relative change was calculated as follows (the FU value − the baseline value/the baseline value)

**Fig. 2:**
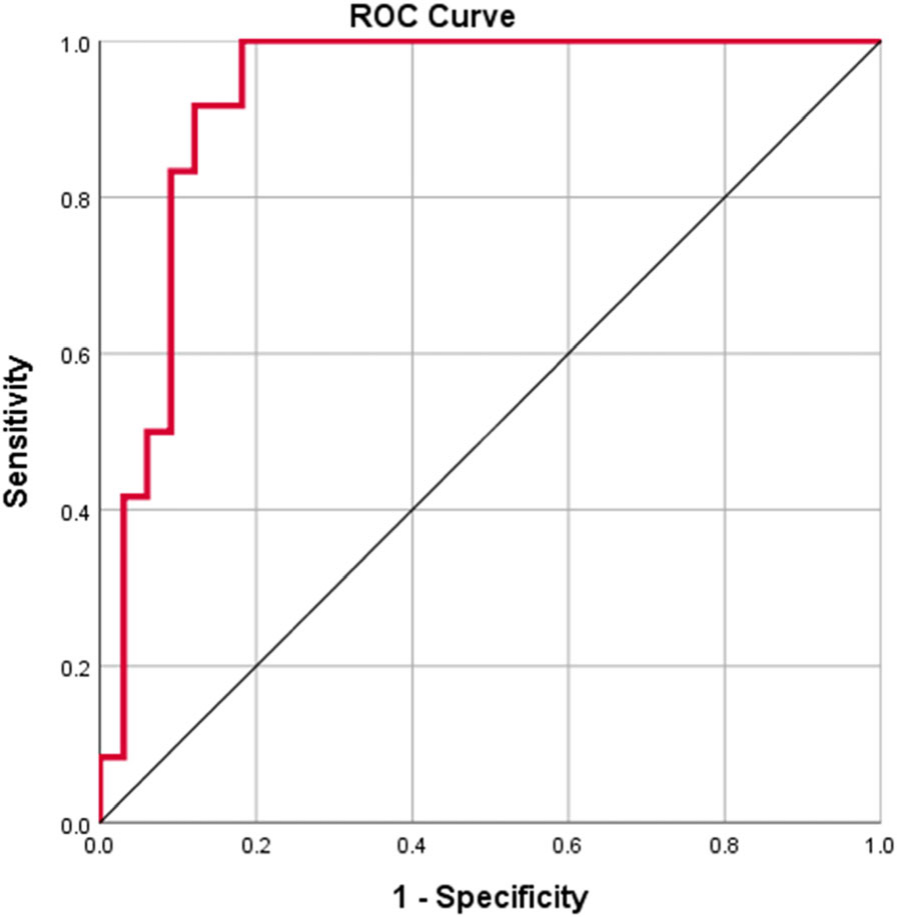
ROC curve analysis of the relative elevation of the NT-proBNP in relation to the relative reduction of the GLS (>15%), AUC = 0.929 and *p* value<0.001

ROC analysis of the 50 patients with elevated follow-up NT-proBNP showed that a 2.2 times elevation of NT-proBNP from the baseline value was able to define a relative reduction of GLS (>15%) by a sensitivity of 100% and a specificity of 81.8% (AUC = 0.929, *p* <0.001) (Fig. 2).

A multivariate regression model with the delta NT-proBNP as the dependent variable showed that the Tei index (*B*= 554.64, *p* < 0.001) was able to predict the change in NT-proBNP level with an adjusted *R* square of 29.2%.

While a multivariate regression model with the delta GLS as the dependent variable showed that the Tei index (*B* = − 8.6, *p* = 0.001), 3D-LVEDV (*B* = 0.167, *p* = 0.02), 3D-LVESV (*B* = − 0.383, *p* = 0.028), and 3D-EF (*B* = − 0.30, *p* = 0.038) were able to predict the change in the GLS with an adjusted *R* square of 21.1%.

None of the other tested clinical or echocardiographic variables showed a predictive relation with either the delta NT-proBNP or the delta GLS.

## Discussion

The large number of cancer survivors increases the likelihood of developing chemotherapy-induced LV dysfunction and overt heart failure. This is especially true in breast cancer female patients where female gender, per se, is a well-established risk factor for cardiotoxicity. In addition, anthracycline, a commonly used chemotherapeutic agent in breast cancer, may generate dose-dependent LV dysfunction, which is associated with poorer prognosis [20].

When patients with chemotherapy-induced cardiomyopathy develop symptoms, the prognosis is one of the poorest in the heart failure population. Yet, those patients remain asymptomatic for a long time [21]. LVEF, which is widely used to monitor cardiac systolic function after chemotherapy, fails to detect subtle alterations in LV function. Once the LVEF has decreased in patients treated with anthracyclines, it may be too late to reverse the course of the cardiomyopathy [22]. More sensitive and specific markers of chemotherapy-induced cardiac dysfunction or myocardial injury may allow for earlier and better adaptations of the oncologic as well as the cardiac treatments. To date, there is no proof of a systematic cardioprotective treatment (angiotensin-converting enzyme inhibitor and/or betablocker) in all cancer patients. However, early cardioprotective treatment in case of subclinical left ventricular dysfunction seems to be promising in the prevention of cardiac events [8].

Global longitudinal strain (GLS) has been shown to be more sensitive than LVEF as a measure of systolic function, and it has been used to identify sub-clinical LV dysfunction in patients with cardiomyopathies [23]. However, the value of these early strain changes in predicting the clinical outcomes is still unknown.

Natriuretic peptides are the most commonly used cardiac biomarkers in HF, and in cardiotoxicity [24]. The ESC Position Paper on cancer treatment and cardiovascular toxicity recommends NT-proBNP for the early detection of cardiac injury and as a marker in the follow-up, as well [11]. Yet, it is unknown whether patients with elevated basal cardiac NT-proBNP have a higher risk of cardiotoxicity, although that they probably have a worse prognosis [25].

A combination of GLS with cardiac biomarkers has been proposed to increase the diagnostic accuracy in detecting early cardiotoxicity. Particularly, the integration of NT-proBNP and LV-GLS could be helpful to identify individual patients who have subtle LV systolic dysfunction. Early recognition and appropriate management of those patients can improve outcomes and decrease progression to overt heart failure [26, 27].

The aim of this study was to assess the value of NT-proBNP in addition to the LV-GLS in detecting early sub clinical ventricular dysfunction (as early as 6 weeks of the first chemotherapy dose) in breast cancer female patients.

The study included 74 middle-aged females with non-metastasizing breast cancer who received 2 types of chemotherapeutic agents, one of them was, mostly, an anthracycline (*n* = 70, 94.6%). The baseline conventional 2D and 3D echocardiographic parameters were normal in all patients. Almost all patients showed normal baseline LV-GLS values (more negative than − 20) except for one patient who had a low baseline LV-GLS (− 16) and that was associated with a high baseline NT-proBNP (459 pg/ml).

Patients came for follow-up, after 6 weeks of the first chemotherapy session, and this gave time for 3 chemotherapy cycles to be completed (the first cycle was given immediately after the first visit, the 2nd cycle was 3 weeks after the first, and the 3rd cycle was 3 weeks later, immediately before the scheduled follow-up visit). All patients came to follow-up, except for one patient who died of unknown etiology just before her scheduled FU visit.

Fifty patients (68.5%) had their NT-pro BNP elevated in the FU visit. They did not experience any heart failure symptoms or signs, yet there was a significant reduction in the 2D and 3D LVEF, as compared to their baseline levels, a finding that was not shown in patients who did not have elevated FU levels of NT-proBNP. In a multivariate analysis model that included all clinical and echocardiographic parameters of the study population, the Tei index was found to be the only predictor of the change in the NT-proBNP plasma level.

Kittiwarawut et al. collected data from 52 female breast cancer patients receiving doxorubicin (mean cumulative dose 237 mg/m^2^) and cyclophosphamide every 3 weeks for four cycles. Cardiac function evaluations by echocardiography and NT-pro BNP were done at baseline and at the end of the fourth cycle of chemotherapy. No symptomatic heart failure was detected during the study period. However, there were significant asymptomatic reductions of left ventricular ejection fraction (LVEF) from a mean of 70.7% at baseline to 67.0% at the follow-up. After chemotherapy, a significant rise of serum NT-proBNP occurred in patients who subsequently developed an LVEF reduction compared with patients with normal LVEF. They concluded that asymptomatic reductions in cardiac function are common in breast cancer patients treated with doxorubicin and that NT-proBNP may serve as a convenient serum biomarker for early detection of cardiotoxicity induced by anthracycline [28]. These findings were also shown by Cil et al., who demonstrated the association between higher NT-proBNP levels and reduced LVEF in asymptomatic breast cancer patients after doxorubicin administration and they stated that this could be an early indication of subclinical acute anthracycline cardiotoxicity. They also concluded that breast cancer patients experiencing a progressive increase in NT-proBNP plasma levels might be at a higher risk for acute anthracycline cardiotoxicity [29].

In this study, only 2 patients had their 2D-LVEF reduced to the guideline proposed definition levels of cardiotoxicity, yet their GLS did not show a significant relative reduction. This could be due to errors in estimation of the EF values or maybe these patients do not simply fit into the guideline’s diagnosis. Patients with myocardial injury causing proBNP elevation could not be individually identified using the conventional echocardiographic LVEF as almost all LVEF values were within the normal range. Upon measuring the 2D LV-GLS absolute values, 22 (44%) patients of those with elevated follow-up levels of NT-proBNP were found to have absolute reduction of the FU LV-GLS to values less than (less negative than) − 20 and 12 patients (24%) had a relative reduction of GLS >15%. This is similar to what Calle et al. [30] had found, as they studied 66 breast cancer patients for early cardiotoxicity associated with anthracyclines-trastuzumab regimen. They found that 13 patients (19.7%) developed early reduction in their LV-GLS, despite normal 2D-LVEF values. In contrast to our study, they followed up their patients for at least 1 year to explore whether the early LV-GLS changes could predict future reduction of LVEF. They concluded that abnormal values of 2D-GLS in the presence of a normal EF can predict a future drop in LVEF. They also recommended that GLS should be used as a marker of stage B heart failure in patients given a combined anthracycline-trastuzumab regimen [30]. In our study, we were only concerned with the early changes of LV function and we did not follow up the patients for the delayed chemotherapy-induced cardiotoxic effects.

A relative reduction in the GLS >15% compared with baseline values was found to be of clinical significance in chemotherapy induced cardiotoxicity [19]. In this study, the median NT-proBNP was significantly higher in patients with a high relative reduction of LV GLS (>15%) versus patients with a less relative reduction. On the other hand, patients with low absolute LV GLS showed a higher, yet not statistically significant, NT-proBNP median values when compared to patients with normal absolute LV GLS. So, the integration between the elevated NT-proBNP and the relative reduction of the LV-GLS was more successful in identifying patients with cardiac injury than the integration between the NT-proBNP and the absolute LV-GLS values. It was also apparent that there is a strong positive correlation between the positive change in the NT-proBNP (the difference between the follow-up and the baseline values) and the relative change in the LV GLS.

A marked elevation of the follow-up NT-pro BNP plasma levels (>200 pg/ml) was found in 5 patients (6.8%). All of them had a lower LV-GLS in the FU visit and 4 of them had a high relative reduction of the LV-GLS (>15%). Neither the 2D-LVEF nor the 3D-LVEF could identify any of these patients as being abnormal.

This study demonstrated the inability of the absolute LVEF values (either 2D or 3D) to detect the early subtle chemotherapy-induced myocardial injury, which was shown by the elevated NT-proBNP values. Even the absolute GLS values were not as accurate as the relative GLS reduction in defining cardiac dysfunction. On the other hand, the association between the relative elevation of the NT-proBNP and the relative reduction of the LV-GLS showed promising results in defining patients with cardiac injury with an excellent sensitivity and a good specificity. Identification of those patients is pertinent as management plans including cardioprotective agents (renin angiotensin blockade and/or beta blockers) could reverse the myocardial injury or at least prevent the progression to cardiac dysfunction.

### Limitations

This study focused only on the short-term outcomes. Future studies should extend the follow-up beyond 6 months for better evaluation of the long-term effects on patients with apparently normal LVEF. We could not include larger sample size because of the restricted financial resources.

## Conclusion

Integration of relative elevation of NT-proBNP plasma level and relative reduction of the 2D LV-GLS may be helpful in the early detection (as early as 6 weeks after the first chemotherapy dose) of chemotherapy-induced cardiotoxicity in asymptomatic breast cancer female patients. The conventional 2D and 3D measurement of LVEF failed to detect these early cardiac abnormalities. Patients who develop a higher relative elevation of NT-proBNP and a higher relative reduction of the LV-GLS (>15%) should be candidates for more meticulous and frequent follow-ups. Early detection of subclinical LV dysfunction will prompt early cardioprotective measures and thus helps to improve the clinical outcomes. Future studies focusing on the role of cardioprotective drugs in patients with subclinical LV dysfunction are recommended.

## Data Availability

The datasets used and/or analyzed during the current study are available from the corresponding author on reasonable request.

## Abbreviations

2D: Two-dimensional
3D: Three-dimensional
BMI: Body mass index
BP: Blood pressure
ECG: Electrocardiogram
EF: Ejection fraction
ELISA: Enzyme-linked immunosorbent assay
ESC: European Society of Cardiology
ET: Ejection time
FS: Fractional shortening
FU: Follow-up
GLS: Global longitudinal strain
Hb: Hemoglobin
HER: Human epidermal growth factor receptor
HF: Heart failure
IVCT: Isovolumetric contraction time
IVRT: Isovolumetric relaxation time
LV: Left ventricle
LVEDV: Left ventricular end-diastolic volume
LVESV: Left ventricular end-systolic volume
MPO: Myeloperoxidase
NT-proBNP: N-terminal brain natriuretic enzyme
PR: Progesterone receptor
SBP: Systolic blood pressure
SD: Standard deviation
STE: Speckle tracking echocardiography
TAPSE: Tricuspid annular plane systolic excursion
TDI: Tissue Doppler imaging
TnT: Troponin T
TTE: Transthoracic echocardiography
TV: Tricuspid valve
